# The function of histone acetylation in cervical cancer development

**DOI:** 10.1042/BSR20190527

**Published:** 2019-04-12

**Authors:** Shanshan Liu, Weiqin Chang, Yuemei Jin, Chunyang Feng, Shuying Wu, Jiaxing He, Tianmin Xu

**Affiliations:** 1 Department of Obstetrics and Gynecology, The Second Hospital of Jilin University, 218 Ziqiang Road, Jilin 130041, P.R. China; 2 Surgical Department, The Second Hospital of Jilin University, 218 Ziqiang Road, Jilin 130041, P.R. China

**Keywords:** Cervical cancer, Epigenetics, Histone acetylation

## Abstract

Cervical cancer is the fourth most common female cancer in the world. It is well known that cervical cancer is closely related to high-risk human papillomavirus (HPV) infection. However, epigenetics has increasingly been recognized for its role in tumorigenesis. Epigenetics refers to changes in gene expression levels based on non-gene sequence changes, primarily through transcription or translation of genes regulation, thus affecting its function and characteristics. Typical post-translational modifications (PTMs) include acetylation, propionylation, butyrylation, malonylation and succinylation, among which the acetylation modification of lysine sites has been studied more clearly so far. The acetylation modification of lysine residues in proteins is involved in many aspects of cellular life activities, including carbon metabolism, transcriptional regulation, amino acid metabolism and so on. In this review, we summarize the latest discoveries on cervical cancer development arising from the aspect of acetylation, especially histone acetylation.

## Introduction

According to statistics, there were 13 240 new cases of cervical cancer and 4170 deaths in the United States in 2018 [1]. High-risk type of human papillomavirus (HPV) infection, especially HPV16 and 18, is closely associated with cervical cancer. Amazingly, 60–90% of other cancers are linked to HPV [2]. HPV is a cyclic DNA virus encoding eight genes in the genome. According to its expression pattern, it can be divided into early (E) or late (L) gene. There are six E genes (e1, e2, e4, e5, e6 and e7) and two L genes (l1 and l2) [3]. The HPV e1 gene is indispensable for the initiation of viral DNA replication. It is expressed in the early stage of virus infection in host cells. E4 is mainly expressed in the late stage of infection, which contributes to the expansion of viral genome, disrupts cytokeratin network and promotes viral release [4]. E2 regulation of host genes may be of great significance in promoting the life cycle of HPV 16. The di-lysines at 111 and 112 are conserved in almost all papillomaviruses. Acetylated K111 enhanced viral replication while deacetylated K111 prevents the helicase activity of e1 and abrogates replication [2]. E5 inhibits the maturation of endocytic vesicles from early to late stage [5]. Viral oncogenes e6 and e7 create an environment to promote cell proliferation and alter the transcription of host genes [6]. The expression of e6 and e7 oncoproteins is no longer inhibited with the absent expression of e1/e2 [7]. E6 and E7 proteins can neutralize the function of p53 and rb members [8]. E6 enhances the accumulations of reactive oxygen species (ROS) induced by tumour necrosis factor (TNF) is a precedent event of E6-enhanced cytolysis. To our surprise, this process is independent of NF-κB activation and p53 [9]. Compared with E6, the HPV16 E6 amino acid 83 variants (aa 83 variants) remarkably enhanced MAPK pathway. It activated Notch signaling and inhibited transformation mediated by Ras. These mechanisms lead to the strong tumorigenicity of aa 83 variants [10]. HPV genes affect chromatin modification in multiple ways, for example e6 and e7 activation induced H3K9 acetylation [11]. Therefore, these oncogenes directly and indirectly affect cell apoptosis, proliferation and growth, leading to tumorigenesis.

Modified proteins are found in cell membranes, cytoplasmic matrix, organelles and nuclei. They are involved in various metabolic reactions, including glycometabolism, tricarboxylic acid cycle and fatty acid metabolism, and are closely related to the activities of living organisms. Lysine acetylation catalyzed by lysine acetyltransferase (KAT) enzymes [12] is known as a kind of post-translational modifications (PTMs), which regulates gene expression by modifying histones and non-histones. Protein acetylation was first identified on histones in the early 1960s, then on non-histone protein more than 20 years later, and was discovered on p53 after another decade. Histone acetylation is one of the fastest PTMs on the aspect of dynamics: faster than histone methylation while slower than phosphorylation [13]. Histone modification is critical to gene regulation. Histone modification can be divided into two types: small chemical group modification and large chemical group modification. Among them, small chemical group modification mainly includes methylation, acetylation, propionylation, butyrylation, phosphorylation, glycosylation, crotonylation and so on. Macrochemical group modification mainly includes ubiquitination, biotinylation, etc. Various acylation modifications occur mainly on the side chains of lysine residues [14]. DNA and five kinds of histones constitute nucleosomes, the basic structural units of chromatin. The tails of histone proteins extend out of the nucleosomes to make the PTM available. Several kinds of PTMs can occur on the same tail and each kind of PTM can have an independent effect. This complex system is maintained by ‘writers’, who deposit the modifications, ‘readers’, who interpret and ‘erasers’, as histone modifications are reversible allowing for genome plasticity [15]. At present, histone modification is usually expressed by a unified Brno nomenclature, and its naming rules are histone name+amino acid residue abbreviation and site+modification type. For example, H4K4me3 represents the trimethylation of the lysine residue at the fourth position of histone H4.

## Histone deacetylases

**Figure 1:**
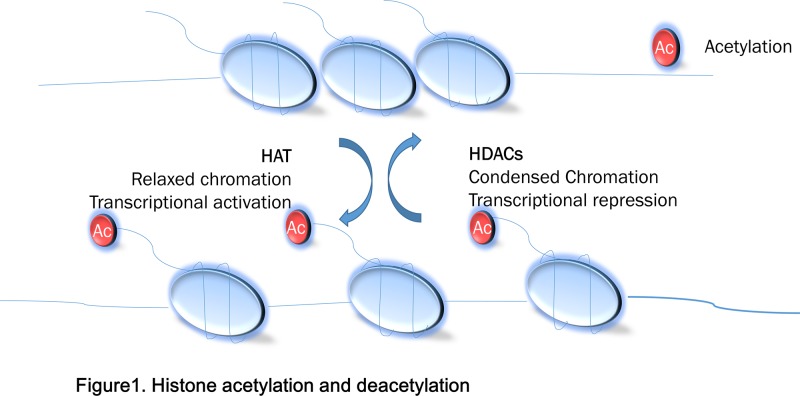
Histone acetylation and deacetylation

Histone acetylation is dynamically and reversibly regulated by histone acetyltransferases (HATs) and histone deacetylases (HDACs). Histone acetylation promotes gene expression by altering the nucleosome’s spatial structure, inducing chromatin loosening, thus increasing gene transcription and replication. HDACs remove acetyl residues and associate with condensed chromatin structures which in turn suppress transcription [16]. The mechanism is illustrated in Figure 1. The families of HDACs, called as eraser enzymes, are sorted into four classes (I–IV). Class I HDACs include HDAC 1, 2, 3 and 8. Class II HDACs include HDAC 4, 5, 6, 7, 9 and 10. Class III, also known as sirtuins/ SIRT1–7, need nicotine adenine dinucleotide as a cofactor. Class IV HDACs refer to HDAC11 [17]. Class I, II, IV are Zn2+-dependent HDACs [18]. Retinoblastoma associated protein 48 (rb ap48) is a component of HDACs and a key mediator for controlling the transformation activity of HPV16 in cervical cancer cells [19]. Because of its significant role in cancer, HDAC protein shave becomes a potential target for cancer treatment. The degree of deacetylation of substances affects their anti-cancer ability [20]. HDAC inhibitor (histone deacetylase inhibitor, HDACI) reduces deacetylation of histones and improves acetylation histone levels by controlling DNA entanglement in histones tightness, therefore playing a role in regulating gene expression. So far, five HDACIs including Chidamide (CS055), Romidepsin (FK228), Panobinostat (LBH589), Belinostat (PXD101) and Vorinostat (SAHA) have been approved for anti-tumor therapy [21]. However, SAHA and other HDACIs have one obvious disadvantage, that is, their production is costly, their toxicity, non-specificity and side effects remain to be concerned [22]. Therefore, it is urgent to find new compounds with good specificity and little side effects.

### Class I HDACs in cervical cancer

HDAC1 mainly distribute in nucleus when transfected alone into HeLa cells. However, co-transfection with ABIN1 (A20 binding inhibitor of NF‐κB1) shifted its location to the cytoplasm. ABIN1 directly binds with HDAC1 to down-regulate HDAC1 ubiquitination and regulates level of p53 via the modulation of HDAC1 level [23]. NF-κB p50 subunit is an important regulator of inflammation and according to recent study it also functions in tumor inhibition. P50 forms homologous isomers to actively inhibit the expression of NF-κB-dependent inflammation genes, and this inhibitory activity of p50 is thought to be partially mediated by interaction with epigenetic inhibitor HDAC1. In human HeLa cells, p50 and HDAC1 immunoprecipitated complexes are found to decrease with inflammatory stimulation. Further studies concluded that HDAC1 may be bound to p50 by physical interactions between two different regions of the nuclear factor-κB subunit. One of these two sites is located at the far end of p50 and has sequence specificity. Mutations in this region result in increasing histone acetylation and expression of cytokines and chemokines [24]. Researchers found that H1K85ac was dynamically regulated in response to DNA damage which in turn reduced its own level. HDAC1 is responsible for the process. The acetylation of the lysine residue at the 85th position of histone H1 regulates chromatin structure and preserves chromosome integrity in case of DNA damage [25]. A research based on HeLa cells and wild-type mouse embryonic stem cells (mESCs) and mESCs with a depletion of HDAC1 found that H3S10ph and gH2AX interact with each other at G2 phase. Because of the radiation-induced damage to the genome, the phosphorylation of H3S10 decreased and the interaction between H3S10ph and gH2AX weakened. Both phosphorylation and acetylation have great effects on radiation-specific changes in the histone signature [26]. H2A.Z-2, a histone variant, is rapidly exchanged at damaged regions after DNA double strand breaks [27]. HDAC 2 and 3 push forward an immense influence on spindle assembly checkpoint (SAC) signaling [28]. The interaction between HDAC3 and PIWIL2 has great effects on HDAC3-mediated epigenetic regulation and cell proliferation on cancer. HDAC3 can also inhibited the function of p53, p27 [29]. HDAC8 not only expressed in the cytoplasm, but also expressed in the nucleolus of HeLa cells. It might be a primary tubulin deacetylase altering the function of alpha tubulin. The hyper acetylation of tubulin can stabilize the microtubules and inhibit cell migration and mitotic phase of cell cycle [30].

### Class II HDACs in cervical cancer

HDAC6 shows cytoplasmic localization. Although named HDAC6, there is no detectable deacetylase activity against histones *in vivo* [31,32]. Its deacetylation activity is regulated by PTM and protein–protein interactions. HDAC6 also associates with microtubules to promote cell motility [33]. Connexin32 (Cx32), a gap junction protein, is widely expressed in various tissues and plays a role in regulating intercellular communication and physiological activities. Inhibition of HDAC6 leads to an accumulation of Cx32 in HeLa cells. Five lysine acetylation targets in the C-terminus of Cx32 have been identified and these relative acetylation of the five lysine played vital functions in controlling cellular proliferation [34].

### Class III HDACs in cervical cancer

The sirtuins, also refer to class III HDACs, contribute to the cellular response to oxidative stress. Multiple sirtuins participate in the regulation of the same biological processes [35]. Hst4 is a member of *Schizosaccharomyces pombe* sirtuin family. According to a recent study, deletion of hst4 caused the delay of S phase of HeLa cells and a defected replication. The mechanism behind this phenomenon is H3K56 acetylation [36]. Using HeLa cells as positive control researchers found that once deacetylation function of sirtuins was suppressed, they were impossible to remove the acetyl groups from arylamine *N*-acetyltransferase 2 (NAT2), making this protein more stable, thereby increasing the activity of NAT2 [37]. The acetylation of P53 increased in Sirt1 silenced HeLa cells. Sirt1-silenced HeLa cells increased resistance to etoposide or hydrogen peroxide therapy by reducing DNA damage, intracellular accumulation of reactive oxygen species and activation of apoptotic pathways [35]. SIRT1 only has impact on the acetylation level but not on the phosphorylation level of Splicing factor SC35 (also name as SRSF2). Further study based on HEK-293FT and HeLa cells revealed that SIRT1 inhibits the SC35-promoted tau exon 10 inclusion by way of changing the acetylation status of SC35. However, the exact deacetylation sites need further research [38]. Besides, Sirt1 controls HPV16 replicate and can be brought to the viral genome by the viral proteins E1 and E2 [39]. SIRT2 modulates cell migration, interacts with heat shock protein 90 (HSP90) regulating its acetylation and ubiquitination in HeLa cells. SIRT2 deacetylates HSP90 and inhibits HSP90/LIMK1/cofilin pathway [40]. In HeLa cells deacetylation of JNK mediated by SIRT2 promotes oxidative stress-induced cell death. Conversely, inhibit SIRT2 decreases H_2_O_2_-mediated cell death [41]. To clarify that infection induces modification of deacetylase SIRT2 to target it to chromatin, HeLa cells and mouse embryonic fibroblasts (MEFs) are used in infection experiments. The phosphorylation state of serine 25 on SIRT2 counts a great deal in Listeria infection, in contrast, dephosphorylation controls its subcellular localization [42]. SIRT2 specifically deacetylates K52 of RhoGDI (a key regulator of Rho proteins, regulating cell signal transduction and energy) and decelerates cervical cancer cell proliferation [43]. SIRT2 also acts as a mitochondrial sirtuin and an autophagy/mitosis regulator to maintain mitochondrial biology, thus promoting cell survival [44]. SIRT3, SIRT4 and SIRT5 are all mitochondrial sirtuins, which are closely associated with tumorigenesis [45]. SIRT5 is known for the characteristics of its mitochondrial localization and preference for negative-charged acyl groups [46], but different tissue has its own character on expression pattern of different isoforms. SIRT5iso1–3 except SIRT5iso4 express in uterus (HeLa), SIRT5iso1 expresses in almost all human tissues, except bladder, adipose and trachea [47]. Previously recorded the activity of SIRT7 *in vitro* was controversial. It has been shown that SIRT7 binds to DNA on the nucleosome through C-terminal base region to enhance its deacylase activity to H3K36/37 [48]. Glycogen synthase kinase (GSK) is a highly conserved serine threonine protein kinase, which is involved in many life activities such as glycogen synthesis, insulin regulation, transcriptional activation and development regulation of various proteins in animals. Acetylation and phosphorylation of GSK3β in HeLa cells over expression SIRT1–SIRT7 [49].

### The HDAC inhibitors

An HDACI usually consists of three domains: (1) a zinc binding group (ZBG); (2) a cap group (CAP); (3) a linker connecting the above two groups [21]. Trichostatin A (TSA), first obtained from streptomyces hygroscopicus, is usually used as the specific inhibitor of HDACs except for class III HDACs [50]. Hypoxia-inducible factor 1 (HIF-1) is an oxygen-sensitive transcription factor which is correlated with tumor metastasis, poor patient prognosis angiogenesis and so on. Study shows TSA acetylated HIF-1α at lysine 674. The acetylation in turn facilitates TSA resistance under normoxic conditions in HeLa cells, suggesting the relationship between acetylation of HIF-1α and drug resistance of cancer therapy [51]. Vorinostat (SAHA) is a PAN-HDACI, which can significantly reduce E6 and E7 activity and selectively induce apoptosis of HPV-infected cells by reducing DNA repair reaction [52]. SAHA is a non-selective HDACI, targeting most of the 11 subtypes of HDAC, which is also responsible for its side effects. This shortcoming can be overcome by modifying at the C4 position of the linker of SAHA. Several modified analogues exhibited high dual HDAC6/8 selectivity which can be used as a kind of chemical tool to clarify the function of HDAC6 and HDAC8 in cancer biology [53]. Oxamflatin is another HDACI. It increase E-cadherin expression in cervical cancer cell lines in a time and concentration dependent way [54]. Valproic acid (VPA), also an HDACI, involves in the re-expression of E-cadherin [55]. *N*-(2′-Hydroxyphenyl)-2-propylpentanamide (OH-VPA) is a VPA derivative with better anti-proliferative effect compared with that of VPA. Study shows that in HeLa cells, OH-VPA acted as both an antioxidant that produces an ROS imbalance and an HDACI [56]. Genistein can restore the expression of tumor-suppressing genes in human cervical cancer cells by inhibiting DNA methyltransferases (DNMTs) and HDACs [57]. HDACI caffeic acid (DHCA) retarded the growth of cervical cell lines. Further studies confirmed that DHCA can bind to HDAC to induce apoptosis [58]. Mocetinostat and entinostat are class I HDACIs with the properties of high efficacy and selectivity in three cervical, six ovarian and two placental cancer cell lines [59]. 2-Oxo-1,3-thiazolidine derivatives are class II HDACIs with good bioactivities, oral bioavailability and ADMET (absorption, distribution, metabolism) toward cervical cancer [22]. Scriptaid (SCR) inhibits HDAC-8 effectively than TSA [60]. 2-Aminobenzamides compound M122 especially inhibits HDAC1 and HDAC2 in six kinds of cancer cell lines [21]. The synthesized isatin-based compounds inhibit HDAC and cellular proliferation of HeLa cells [61]. We summarize the details of HDACIs mentioned above in Table 1.

**Table 1:** Summary of HDACIs in cervical cancer recently

Name	Type	Property	Disease or cell	Reference
2-Aminobenzamides compound M122	Especially inhibit HDAC1 HDAC2	High potency and selectivity	HeLa, etc.	[21]
2-Oxo-1,3-thiazolidine derivatives	Class II HDACIs	Good bioactivities, oral bioavailability	Cervical cancer	[22]
TSA	Except for class III HDACs	Acetylated HIF-1α at lysine 674	HeLa cells	[50,51]
Vorinostat (SAHA)	Pan-HDAC inhibitor	Reduce E6 and E7 activity	HPV infections	[52,53]
Oxamflatin	HDAC inhibitor	Induces E-cadherin Expression	HeLa cells	[54]
Valproic acid (VPA)	HDAC inhibitor	Re-expression of E-cadherin	HeLa and TC1 cell lines	[55]
OH-VPA	HDAC inhibitor	Antioxidant	HeLa cells	[56]
Genistein	HDAC families	Time-dependent	HeLa cells	[57]
Caffeic acid	HDAC inhibitor	Induction of apoptosis	Colon and cervical cancer cells	[58]
Mocetinostat and entinostat	Class I HDACIs	Pan-gynecologic cancer inhibitors	Ovarian, cervical cells, etc.	[59]
Scriptaid (SCR)	HDAC-8 inhibitor	Inhibit HDAC-8 effectively than TSA	HeLa cells, etc.	[60]
Isatin-based compounds	HDACIs	Inhibit proliferation	HeLa cells	[61]

## Histone acetyltransferases (HATs)

There are two major types of HAT according to the location, nuclear type (A-type) and cytoplasmic type (B-type) [62]. The MYST (Moz, Ybf2/Sas3, Sas2, Tip60) family, the GCN5-related *N*-acetyltransferases (GNAT) family and the p300/CREB-binding protein (CBP/CREBBP) family are the subfamilies of A-type, as they have structural homologies and functional similarities. Type-B HATs are categorized by acetylating newly synthesized histone H3 and H4 [17]. The MYST family contains five members: KAT6A traditionally called MOZ (monocytic leukemic zinc-finger protein) or MYST3; KAT6B, also known as MORF (MOZ-related factor) or MYST4; KAT7, named HBO1 or MYST2; KAT8, referred to as hMOF or MYST1; and KAT5 (or Tip60) [12]. The MYST-ing complex contains different subunits, which carry different histone recognition modules (chromatin, bromine and PWP domains) to combine different PTM residues [63]. Histone acetyltransferases can also acetylate non-histones, including cancer-promoting and anti-cancer factors.

### The MYST family

Human MOF (HMOF) (males absent on the first) proteins belong to the MYST family of histone acetyltransferases, specifically acetylate histone H4 at lysine 16 (H4K16ac), play a vital role in DNA damage response and transcription [64]. Knockout of HMOF in HeLa cells results in a prominent reduction in acetylation of histone H4K16 [65]. In several stages of DNA damage response, MOF plays a crucial role in inhibiting replication stress induced by genotoxic factors. In HeLa cells, after removing MOF, cell viability is significantly reduced after treatment with three genotoxic drugs (cisplatin, hydroxyurea and camptothecin) [66]. In addition to the nucleus, MOF residing in mitochondria can rescue respiratory defects in HeLa cells [67]. What’s more, it has been also identified as an evolutionarily conserved histone crotonyltransferase [68]. MORF (monocytic leukemia zinc-finger protein-related factor, also known as KAT6B or MYST4) DPF domain is a reader of global H3K14 acylation [69]. HIV1-TAT interactive protein (TIP60) is a tumor suppressor mediating growth suppression of cervical cancer [70]. Researchers prove that TIP60 regulates the interaction between NDC80 (a tetramer composed of centromere protein HEC1) and spindle microtubules during the process of mitosis, and acetylates HEC1 on two evolutionally conservative residues, Lys-53 and Lys-59. This acetylate activity weakens the phosphorylation of HEC11-80 at Ser-55 and Ser-62 [71]. TIP60/TRRAP histone acetylase complex participates in e2-mediated inhibition [72]. When TIP60 was overexpressed, the expression of HPV18 e6 and e7 genes was significantly inhibited. In addition, the co-overexpression of TIP60 and ep400 in HeLa cells synergistically inhibited the expression of HPV18 e6-e7 [73]. Overexpression of TIP60 reduces the DNA methylation levels in HeLa cells [74]. TIP60/TRRAP complex also promotes oxidative stress resistance by up-regulating the expression of FoxO transcription factor in HeLa cells. The acetylation level of H4K16 was increased by recruiting TIP60 into the promoter region of Fox1 gene [75]. The binding of the bromodomain protein BRD2 with H4Ac protects acetylated chromatin from HDAC and allows acetylation to spread along the flanking chromatin [76]. BRD4 which has been shown to regulate HPV infection is a member of the bromine domain (BRD) family protein. It serves as a ‘reader’ to recognize acetylated lysine, thereby promoting chromatin remodeling and transcriptional activity [77].

### The GCN5-related *N*-acetyltransferases family

Lysine acetyltransferase GCN5 was up-regulated in cells expressing HPV E7 protein. Knockout of GCN5 can reduce the stability of transcription factor E2F1. Loss of E2F1 results in G1 phase arrest. The high expression of E2F1 saved the inhibition of GCN5 knockout on the G1/S process of HPV E7 expressing cells. CHIP studies have shown that GCN5 binds to E2F1 promoter and increases the degree of histone acetylation in the region. These results revealed the new mechanism of lysine acetyltransferase GCN5 in the pathogenesis of cervical cancer, that is, lysine acetyltransferase GCN5 promotes the cell proliferation induced by HPV E7 by up-regulation of E2F1 [78]. Eighty percent of all human proteins receive an acetyl group at their N-terminals. N-terminal acetyltransferases (NATs) modify the N-terminal of proteins by catalyzing the transfer of acetyl groups to amino groups in the main chain of proteins. There are six known human NATs, which named from NATA to NATF according to the type of acetyltransferase activity [79]. The activity of NAT is mediated by the catalytic NAT *N*-acetyltransferase (Naa) subunits Naa10 to Naa60. Acetylation of NAT10 at K426 is required for activation of rRNA transcription [80]. Naa30 is one of the components of Nat C complex. Overexpression of Naa30362 in HeLa cells can improve cell viability [81]. Naa60 is a peripheral membrane protein and has membrane-binding ability to facilitate itself anchoring to the Golgi apparatus of HeLa cells [82]. NAA80, immunoprecipitated from cervical cells, is an Nt-acetylating actin. Further study reveals that NAA80-knockout cells display seriously altered cytoskeletal organization and dynamics [83]. According to a large-scale reanalysis of experimental data on the HeLa cancer cell line, a new ‘missing protein’ was observed and 392 new protein N-terminal acetylation sites could be identified [84].

### The p300/CREB-binding protein family

P300 and CBP are also responsible for histone crotonylation, acting as the histone crotonyltransferase of H3 with the highest activities [68,85]. The homeostatic sensor mTORC1 acts as a direct activator of p300, and mTORC1-p300 pathway plays a key role in cell metabolism [86].

## Acetylation in mitosis

Chromatin aggregations, nuclear membrane ruptures and transcription decreases during mitosis. The interphase chromatin structure is reconstructed, and transcription resumes immediately after mitosis. The reconstruction of interphase chromatin may be accomplished by ‘bookmarking’, and histone modifications may play a role in this process [87]. Separase is an evolutionary conserved protease, which properly triggered the sister chromatid separation. Its depletion in HeLa caused a strong acetylation of cohesion’s structural maintenance of chromosomes protein 3 (SMC3) subunit and speeds up replication fork [88]. Microtubules are the key components of the cytoskeleton. As highly dynamic polymers, they aggregate and decompose in a short time. Besides cell division and mitosis, microtubules play an important role in maintaining cell shape, intracellular transport, cell movement and signal transduction. Microtubulin (tubulin) is the main protein that makes up microtubules. Microtubulin has two nucleotide (GTP) binding sites and three drug binding sites [89]. The deregulation of mitotic microtubule (mt) dynamics leads to spindle assembly defects and chromosome mismatches, which further lead to chromosome instability, which is a sign of cancer cells [90]. Mutations of the SPG4 (SPAST) gene are the main causes of hereditary spastic paraplegia. In cervical cancer cells two isoforms of spastin protein encoded by SPG4 harboring the same missense mutation bind and bundle different subsets of microtubules, which can be stabilized by increasing the level of acetylated tubulin [91]. Triphala (Trl) inhibits proliferation and suppresses the clonogenicity of HeLa cells. Perturbation of tubulin and its acetylation in cells is the possible anti-proliferation mechanism of Trl [92]. As we know acetylation of tubulin can reduce its nucleation potential [93], safranal, a kind of traditional Chinese medicine, does not induce tubulin acetylation but disrupts the secondary structure of tubulin. It inhibits HeLa cell viability depending on concentration, with minimal damage to cellular microtubules [94]. The mitotic kinesin Eg5 is a member of the kinesin superfamily. K146Q is a pseudo acetylation mutant of Eg5. The expression of the K146Q acetylation mimetic reduces spindle pole separation velocity in mitotically active cells [95]. Pygopus (Pygo)2 plays a significant role in mitotic segregation of chromosomes both in SKOV-3 cells and in HeLa S3 cells, its chromatin effector up-regulating myc-dependent mitotic gene expression. Pygo2 maintains histone H3K27 acetylation and enriches at promoters of biorientation and segmentation genes [96]. A study based on HeLa cells and various mouse tissues reveals that TGF-beta-activated kinase 1 (TAK1) directly binds to αTAT1 to enhance acetylation of microtubules. Recently, it has been shown that TAK1 selectively inhibits AKT through acetylation of microtubules, and further inhibits mitogenic and metabolism-related pathways [97].

## Acetylation and drugs

The 6-phosphofructo-2-kinase/fructose-2,6-biphosphatase 3 (PFKFB3), uniquely localized in the nucleusisa potent allosteric stimulator of glycolysis. The researchers found that the PFKFB3 inhibitor PFK15 alone had no significant effect on HeLa cell apoptosis, but significantly enhanced cisplatin-induced apoptosis. The loss of PFKFB3 also activated the apoptotic effect of HeLa cells on cisplatin-induced apoptosis. Together, acetylation accumulates PFKFB3 in cytoplasm, which in turn promotes glycolysis and protects cells from cisplatin-induced apoptosis [98]. The combination of graphene oxide and cisplatin (GO/CDDP) elicits cervical cancer cells’ death with CDDP and histone H1/H4 co-migrating into the nucleus. GO/CDDP treatment enhanced H4K16ac in the nucleus. This activity results in chromatin relaxation, therefore triggering DNA cleavage and cell death [99]. TIPE1 is a member of the TIPE family functioning in cell proliferation, immunity and carcinogenesis. It enhances cervical cancer proliferation by reducing p53 acetylation and is also related to poor prognosis of cervical cancer and cisplatin resistance [100].

## New method

New molecular technologies are contributing to these discoveries. Requiring no chemical derivatizations, being digested either in-solution or in-gel, and analyzing histones from crude preparations when only small samples are available, are the features of the neprosin-generated method. More than 200 proteoforms can be detected in a single analysis of histones from HeLa S3 cells, which represents the method to be a useful new addition to effective histone analysis [101]. ENCHANT is an improved method of selective proteomics, which can complement the traditional shotgun method in qualitative and quantitative proteomics research [102]. Ultra-high-performance liquid chromatography–mass spectrometry (UHPLC–MS) can simultaneously measure the activity of HDAC1 and HDAC6 in HeLa Cells [103].

## Conclusion and perspective

Except for the above functions, acetylation interacts with methylation, ubiquitinates, autophagy [70,104] and succinylation [105]. Since cervical cancer formation is correlated to high risks of HPV infection, HPV vaccine can prevent women from HPV infection, but what about those already infected? We know that early cervical cancer screening has a certain rate of missed diagnosis. Radiotherapy and chemotherapy used for treatment also bring great pain to patients, as well as drug resistance and radiotherapy resistance that cannot be ignored. Although virus infection cannot be reversed easily, epigenetic changes are reversible enough and thus are able to be regulated. Acetylation affects the function of organisms. The disorder of acetylation regulation is closely related to many diseases. In the past decade, great progress has been made in the research and development of drugs targeting DNA methylation and demethylation, histone acetylation and deacetylation, histone acetylation recognition and disease-related enzymes and protein complexes. Many small molecule drugs have been used in clinical trials to treat cancer and other diseases. Combining epigenetic genomics, proteomics and other modern technologies, large data analysis of diseases is expected to accurately diagnose the possible pathogenesis of individual patients, so as to carry out precise treatment. Although the road is long and tortuous, the technology and therapy based on epigenetics may bring new ideas to the treatment of cervical cancer.

## Abbreviations

Cx32: connexin32
DHCA: caffeic acid
GNAT: GCN5-related *N*-acetyltransferases
GSK: glycogen synthase kinase
HAT: histone acetyltransferase
HDAC: histone deacetylase
HIF-1: hypoxia-inducible factor 1
HMOF: human MOF
HPV: human papillomavirus
HSP90: heat shock protein 90
MOF: males absent on the first
MORF: MOZ-related factor
MOZ: monocytic leukemic zinc-finger protein
MYST: Moz, Ybf2/Sas3, Sas2, Tip60
NAT: *N*-acetyltransferase
OH-VPA: *N*-(2′-hydroxyphenyl)-2-propylpentanamide
PTM: post-translational modifications
ROS: reactive oxygen species
SCR: scriptaid
TAK: TGF-beta-activated kinase
TIP60: HIV1-TAT interactive protein
TSA: trichostatin A
VPA: valproic acid
